# GPS-ARM: Computational Analysis of the APC/C Recognition Motif by Predicting D-Boxes and KEN-Boxes

**DOI:** 10.1371/journal.pone.0034370

**Published:** 2012-03-29

**Authors:** Zexian Liu, Fang Yuan, Jian Ren, Jun Cao, Yanhong Zhou, Qing Yang, Yu Xue

**Affiliations:** 1 Hubei Bioinformatics and Molecular Imaging Key Laboratory, Department of Systems Biology, College of Life Science and Technology, Huazhong University of Science and Technology, Wuhan, Hubei, China; 2 Hefei National Laboratory for Physical Sciences at Microscale and School of Life Sciences, University of Science and Technology of China, Hefei, China; 3 State Key Laboratory of Biocontrol, School of Life Sciences, Sun Yat-sen University, Guangzhou, Guangdong, China; University College Dublin, Ireland

## Abstract

Anaphase-promoting complex/cyclosome (APC/C), an E3 ubiquitin ligase incorporated with Cdh1 and/or Cdc20 recognizes and interacts with specific substrates, and faithfully orchestrates the proper cell cycle events by targeting proteins for proteasomal degradation. Experimental identification of APC/C substrates is largely dependent on the discovery of APC/C recognition motifs, e.g., the D-box and KEN-box. Although a number of either stringent or loosely defined motifs proposed, these motif patterns are only of limited use due to their insufficient powers of prediction. We report the development of a novel GPS-ARM software package which is useful for the prediction of D-boxes and KEN-boxes in proteins. Using experimentally identified D-boxes and KEN-boxes as the training data sets, a previously developed GPS (Group-based Prediction System) algorithm was adopted. By extensive evaluation and comparison, the GPS-ARM performance was found to be much better than the one using simple motifs. With this powerful tool, we predicted 4,841 potential D-boxes in 3,832 proteins and 1,632 potential KEN-boxes in 1,403 proteins from *H. sapiens*, while further statistical analysis suggested that both the D-box and KEN-box proteins are involved in a broad spectrum of biological processes beyond the cell cycle. In addition, with the co-localization information, we predicted hundreds of mitosis-specific APC/C substrates with high confidence. As the first computational tool for the prediction of APC/C-mediated degradation, GPS-ARM is a useful tool for information to be used in further experimental investigations. The GPS-ARM is freely accessible for academic researchers at: http://arm.biocuckoo.org.

## Introduction

The 2001 Noble Prize in Physiology or Medicine was awarded to Leland Hartwell, Paul Nurse and Timothy Hunt for their seminal discoveries of key regulators of the cyclin-dependent kinases (CDKs) which are active in the cell cycle and cellular proliferation [1], [2]. Besides CDK-mediated phosphorylation, cell cycle proteins are modulated by other mechanisms, such as ubiquitin-dependent degradation, which is mainly mediated by the Skp1-cullin-F box (SCF) and the APC/C [3]–[8]. As a high-molecular-mass complex composed of 13 core subunits [3], [5], APC/C was first identified as an E3 ligase for the degradation of mitotic cyclins [9]. Beyond mitosis, APC/C-mediated degradation also plays an important role in regulating Rho GTPase activity [10], [11], axon growth [12], cell adhesion [13] and glycolysis [14], [15]. In this regard, the identification of APC/C-specific degradation substrates is fundamental to understanding the molecular mechanisms and regulatory roles of APC/C.

In 1991, Glotzer *et al.* first characterized an ennea-peptide (9aa) located at the N-terminus of cyclin B which is responsible for its degradation during mitotic exit [16]. Further analyses revealed that the destruction box or D-box follows a minimal consensus of R*XX*L (where *X* is any amino acid), while two co-activators of APC/C, Cdh1 and Cdc20, directly target and interact with the D-box [17]–[19]. Recently, a structural analysis revealed that a core APC/C subunit of Apc10 can also interact with the D-box and contribute to recognition specificity together with Cdh1 [20]. A second APC/C degron, the KEN-box motif with a consensus sequence of KEN, is recognized by Cdh1 and Cdc20 [21], [22]. Although a number of non-canonical destruction signals were experimentally identified, such as the A-box (QRVL) of Aurora-B kinase [23], the G*X*EN motif in *Xenopus* chromokinesin Kid (Xkid) [24], the CRY-box in mammalian Cdc20 [25] and so on, the D-box and the KEN-box are still regarded as the major APC/C recognition motifs [3]–[6], [26].

Conventional experimental identification of APC/C targets using a site-directed mutagenesis strategy is time-consuming, labor-intensive and inefficient [16]–[18], [20]. Although many experimental efforts have been undertaken over the past two decades, the number of known APC/C substrates is still quite limited. In contrast with the experimental approaches, computational prediction and analysis of the D-box and the KEN-box proteins can generate useful information for further experimental manipulation. Recently, the SLiMSearch 2.0 web server was developed for identifying user-defined short linear Motif in a proteome, using evolutionary conservation and protein structural disorder context to score occurrences [27]. However, it is evident that that the prediction with the two loosely defined motifs of R*XX*L and KEN will most likely generate too many false positive hits, suggesting that more sophisticated approaches are needed. For example, Michael *et al.* predicted 25 KEN-box proteins as potentially new APC/C targets by means of a combination of the enrichment of the cell cycle Gene Ontology (GO) terms together with native disorder prediction and motif conservation information [28]. However, only four known APC/C substrates were included in their results (CycA, P14785; KIF22, Q14807; BUB1B, O60566; PDS1, P40316. See in Table S1). In this regard, the development of a general and efficient predictor for D-boxes and KEN-boxes is urgently needed.

In this work, we developed a novel GPS-ARM software package for the prediction of potentially functional D-boxes and KEN-boxes in APC/C substrates. The experimental data was collected from the scientific literature, while the previously developed GPS 2.2 algorithm was adopted for training and prediction. By extensive evaluations, the prediction performance of GPS-ARM determined to be promising and much better than using simple short motifs. With this powerful tool, we systematically analyzed the functional abundance and diversity of D-box and KEN-box proteins in *H. sapiens*. From the results, it is evident that KEN-box proteins are the ones more clearly implicated in cell cycle and mitosis, while both the D-box and KEN-box proteins regulate a variety of biological processes in addition to the cell cycle. Moreover, with additional co-localization information, we predicted hundreds of mitosis-specific D-box and KEN-box proteins in eukaryotes with high confidence. Taken together, the prediction and analysis results are helpful for further experimental consideration, and the GPS-ARM can serve as a useful program for experimentalists. The online service and local packages of GPS-ARM 1.0 were implemented in JAVA and could be freely accessed for academic research at: http://arm.biocuckoo.org.

## Materials and Methods

### Data preparation

We searched the PubMed database with the keywords of “D box” and “KEN box”, followed by a review of the scientific literature published before August 16^th^, 2011. The non-canonical motifs were discarded, while the collected D-boxes and KEN-boxes were required to follow the consensus motifs of R*XX*L and KEN, respectively. In total, we obtained a non-redundant dataset with 74 experimentally identified D-boxes in 68 unique proteins and 44 known KEN-boxes in 42 APC/C substrates (Table S1). The corresponding sequences of these proteins were retrieved from the UniProt database.

Here, we defined an *APC/C recognition motif* ARM(*m, n*) as a core motif of R*XX*L (for the D-box) or KEN (for the KEN-box) flanked by *m* amino acids upstream and *n* amino acids downstream. As previously described [29], all experimentally verified D-boxes or KEN-boxes were regarded as positive data(+), while all other ARM(*m, n*) peptides in the same proteins were taken as negative data(−). Ultimately 217 and 16 negative peptides were obtained for the D-box and the KEN-box, respectively.

For proteome-wide analysis, we also downloaded 6,620, 3,334, 3,124, 16,384 and 20,245 reviewed protein sequences of *S. cerevisiae*, *C. elegans*, *D. melanogaster*, *M. musculus* and *H. sapiens*, respectively, from the UniProt database.

### Performance evaluation

As previously described [29], we used the five measurements of accuracy (*Ac*), precision (*Pr*), sensitivity (*Sn*), specificity (*Sp*) and Mathew's Correlation Coefficient (*MCC*) to evaluate the prediction performance. The measurements were defined as below:
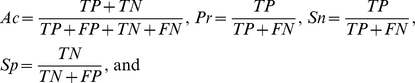


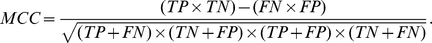
 In this work, the leave-one-out (LOO) validation and 4-, 6-, 8- and 10-fold cross-validations were performed. The Receiver Operating Characteristic (ROC) curves were drawn, and AROC (area under ROC) values were calculated.

### The algorithm

Recently, we developed the GPS 2.2 algorithm mainly for the prediction of protein pupylation sites in prokaryotes [29]. In this work, that algorithm was adopted and described as below.

The GPS 2.2 algorithm comprises two major parts, scoring strategy and performance improvement. In the former step, based on the hypothesis that similar short peptides exhibit similar biochemical properties and biological functions [29], it is possible to use an amino acid substitution matrix, e.g., BLOSUM62, to calculate the similarity between the two ARM(*m*, *n*) peptides of *A* and *B* as below:
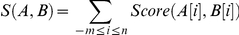
The *Score*(*A*[*i*], *B*[*i*]) represents the substitution score of the two amino acids of *A*[*i*] and *B*[*i*] in the amino acid substitution matrix. If S(*A*, *B*)<0, we simply redefined it as S(*A*, *B*) = 0.

The second procedure comprises the three sequential steps of motif length selection (MLS), weight training (WT) and matrix mutation (MaM). To evaluate the performance improvement, we fixed the *Sp* at 90% and compared the *Sn* values of the LOO validation.

1. **Motif length selection (MLS)**. In this step, the combinations of ARM(*m*, *n*) (*m* = 1, …, 30; *n* = 1, …, 30) were extensively tested, while the optimized combination of ARM(*m*, *n*) was determined to have the highest LOO performance.
2. **Weight training (WT)**. Since different positions can generate different contributions to recognition specificity, we can refine the substitution score between the two ARM(*m*, *n*) peptides A and B can be refined as:
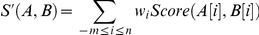
 Here *w_i_* is the weight of position *i*. Again, if *S′*(*A*, *B*)<0, we simply redefined it as *S′*(*A*, *B*) = 0. Initially, *w* was defined as 1 for each position. We randomly picked out the weight of any position for +1 or −1, and adopted the manipulation if the LOO performance was increased. The process was repeated until convergence was attained.
3. **Matrix mutation (MaM)**. BLOSUM62 was chosen as the initial matrix and the leave-one-out performance was calculated. Subsequently, we improved the performance by randomly picking out an element of the matrix for +1 or −1. The procedure was terminated when the performance was not increased any further.

### Implementation of the online service and local packages

The online service and local packages of GPS-ARM 1.0 were implemented in JAVA and are freely available at http://arm.biocuckoo.org/. For the online service, we tested GPS-ARM 1.0 on a variety of internet browsers, including Internet Explorer 8.0 and Mozilla Firefox 7.0.1 under the Windows XP Operating System (OS), Mozilla Firefox 7.0 under Fedora Core 6 OS (Linux), and Safari 3.0 under Apple Mac OS X 10.4 (Tiger) and 10.5 (Leopard). For the Windows and Linux systems, the latest version of Java Runtime Environment (JRE) package (JAVA 1.4.2 or later versions) should be pre-installed. However, for Mac OS, GPS-ARM 1.0 can be directly used without any additional packages. For convenience, we also developed local packages of GPS-ARM 1.0 which support the three major Operating Systems Windows, Linux and Mac.

## Results

### Development of GPS-ARM for the prediction of the D-boxes and KEN-boxes

Although not true in all cases, most of the APC/C substrates contain the D-box and/or the KEN-box, which can be recognized and interact with Cdh1 and/or Cdc20 as two major APC/C recognition motifs [3]–[6], [26]. Since the core motifs were too short and not stringent, extended consensus sequences were utilized, such as R*XX*L*XX*-I/V-*X*N (Motif-D1) [3], R*XX*L*XXXX*N (Motif-D2) [4], [5], [16], and R*XX*L*XX*-L/I/V/M (Motif-D3, from the Eukaryotic Linear Motif resource) [30] for the D-box, and KEN*XXX*-N/D (Motif-KEN) [3], [22] for the KEN-box. However, in our dataset, only 9, 18, 41 and 7 boxes follow the patterns of Motif-D1, Motif-D2, Motif-D3 and Motif-KEN, respectively (Table S1). Thus, these motifs can not be used as predictive indicators due to a low sensitivity.

**Figure 1:**
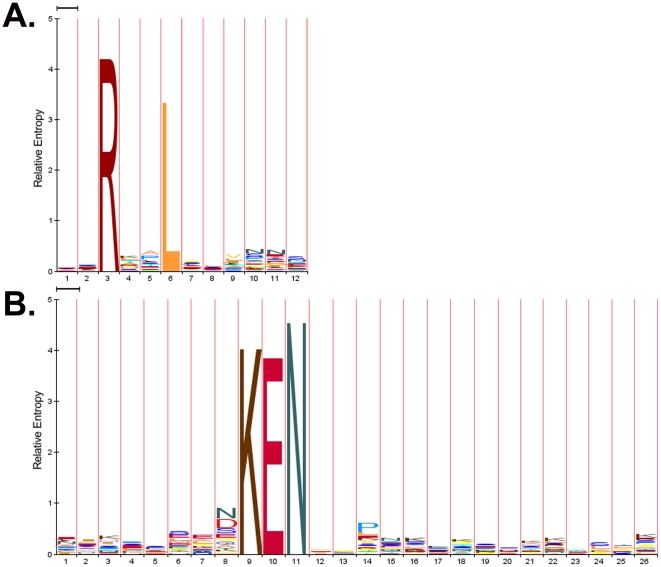
The Sequence logos of ARM(2, 6) and ARM(8, 15) were generated by the HMM-Logo (LogoMat-M) [**31**] for the (A) D-box and (B) KEN-box, respectively.

In this work, we hypothesized that flanking sequences around R*XX*L and KEN could contribute additional specificity for APC/C recognition. In this regard, a recently developed GPS 2.2 algorithm [29] was used for training and predicting. The ARM(2, 6) and ARM(8, 15) were determined as the optimal motifs of the D-box and KEN-box, respectively. To strengthen our hypothesis, the sequence logos of ARM(2, 6) and ARM(8, 15) were created by the HMM-Logo [31] for the D-box (Figure 1A) and KEN-box (Figure 1B), respectively. For the D-box, amino acid residues of V, N, and N preferentially appear at positions of +3, +4 and +5, although weakly (Figure 1A). For the KEN-box, the N residue is weakly informative at positions of +4 (Figure 1B). Thus, the features of known motifs were largely included in the computational models of GPS-ARM. Also, we observed that the amino acids located in R*XX*L are also weakly informative (Figure 1A), whereas residues of N/D and P are moderately informative at positions of −1 and +3 for the KEN-box (Figure 1B). In this regard, our models contained more useful information than known simple motifs.

**Figure 2:**
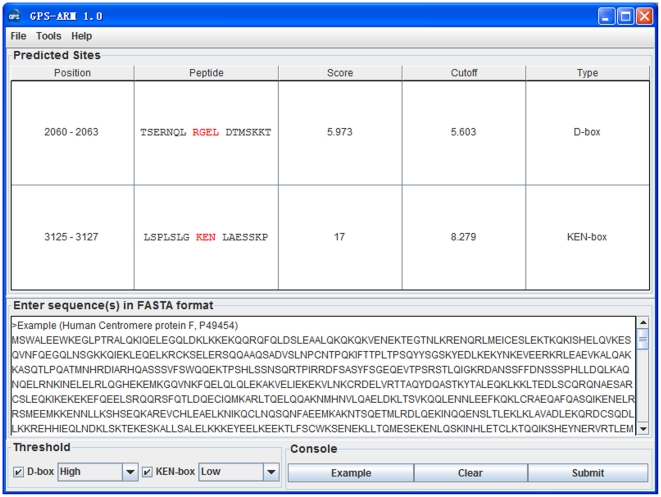
Screen snapshot of the GPS-ARM 1.0 software. The default thresholds were chosen for the D-box (high) and KEN-box (low). As an example, the prediction results for the human centromere protein F/CENP-F (UniProt ID: P49454) are shown.

The software packages of GPS-ARM 1.0 were implemented, and the ARM(7, 7) is shown for convenience. As an example, the prediction results of human centromere protein F/CENP-F (UniProt ID: P49454) was shown (Figure 2). Although human CENP-F is a large protein (3210aa) with up to eight putative KEN-boxes, experimental analysis of its C-terminal fragment (630aa) revealed that disruption of a single KEN-box (3125–3127) is sufficient to inhibit degradation [21]. In these results, this motif was correctly predicted as the only positive hit, while an additionally predicted D-box (RGEL, 2060–2063) should prove useful for further experimental verification (Figure 2).

### Performance evaluation and comparison

**Figure 3:**
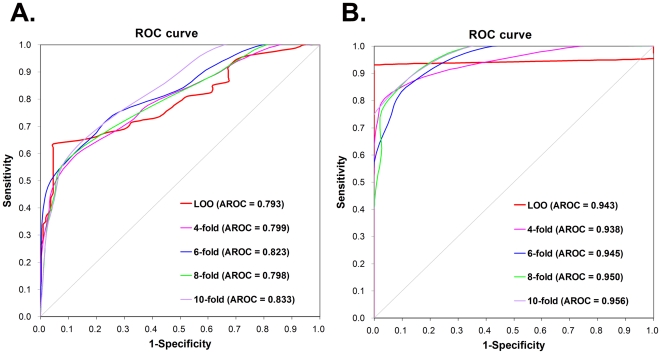
The prediction performance of GPS-ARM 1.0. The LOO validation and 4-, 6-, 8- and 10-fold cross-validations were performed for (A) the D-box and (B) the KEN-box.

To evaluate the prediction performance and robustness of GPS-ARM, LOO validation and 4-, 6-, 8-, 10-fold cross-validations were performed (Figure 3). ROC curves were drawn, while the AROC values were 0.793 (LOO), 0.799 (4-fold), 0.823 (6-fold), 0.798 (8-fold) and 0.833 (10-fold) for the D-box (Figure 3A), and 0.943 (LOO), 0.938 (4-fold), 0.945 (6-fold), 0.950 (8-fold) and 0.956 (10-fold) for the KEN-box (Figure 3B). Since the results of the 4-, 6-, 8- and 10-fold cross-validations were similar to the LOO validation, GPS-ARM 1.0 is evidently a stable and robust predictor. The performance of the LOO validation was also used for the cut-off setting and further comparison, and the three thresholds of high, medium and low were selected (Table 1). In addition, given the highest *MCC* values, the high (0.6463) and low (0.8858) thresholds were chosen as the default thresholds of the D-box and KEN-box, respectively (Table 1).

**Table 1:** Performance evaluation and comparison of the GPS-ARM with known motifs.

Method	Threshold	*Ac*(%)	*Pr*(%)	*Sn*(%)	*Sp*(%)	*MCC*
D-box (GPS-ARM)	High	87.29	82.46	63.51	95.39	0.6463
	Medium	80.76	61.54	64.86	86.18	0.5018
	Low	76.63	53.26	66.22	80.18	0.4346
	e	81.10	95.24	27.03	99.54	0.4471
KEN-box (GPS-ARM)	High	86.67	100.00	81.82	100.00	0.7385
	Medium	91.67	100.00	88.64	100.00	0.8218
	Low	95.00	100.00	93.18	100.00	0.8858
Motif-D1^a^		77.32	90.00	12.16	99.54	0.2797
Motif-D2^b^		75.95	56.25	24.32	93.55	0.2488
Motif-D3^c^		76.29	53.25	55.41	83.41	0.3832
Motif-KEN^d^		38.33	100.00	15.91	100.00	0.2192

For the construction of the GPS-ARM software package, the three thresholds of high, medium and low were selected for D-box and KEN-box, respectively.

a Motif-D1, R*XX*L*XX*-I/V-*X*N [3];

b Motif-D2, R*XX*L*XXXX*N [4], [5], [16];

c Motif-D3, R*XX*L*XX*-L/I/V/M [30];

d Motif-KEN, KEN*XXX*-N/D [3], [22];

e For comparison, we fixed the *Sp* value of GPS-ARM so as to be identical with Motif-D1.

To clearly demonstrate the superiority of GPS-ARM, we also calculated the performances of Motif-D1 [3], Motif-D2 [4], [5], [16], Motif-D3 [30] and Motif-KEN [3], [22] (Table 1). For the D-box, we fixed the GPS-ARM *Sp* value to be identical with that of Motif-D1 (Table 1). By comparison, the *Sn* value of GPS-ARM is much larger than that of Motif-D1 (27.03% vs. 12.16%) (Table 1). Also, the performance of GPS-ARM is better than Motif-D2, since both the *Sn* and *Sp* scores are higher (*Sn*: 27.03% vs. 24.32%; *Sp*: 99.54% vs. 93.55%) (Table 1). Furthermore, both the *Sn* and *Sp* values of GPS-ARM are much better than Motif-D3 (*Sn*: 64.86% vs. 55.41%; *Sp*: 86.18% vs. 83.41%) (Table 1). For the KEN-box, although the *Sp* score of Motif-KEN can reach as high as 100%, its *Sn* value is much lower (Table 1). In addition, we compared the prediction results of GPS-ARM and various motifs for five eukaryotic proteomes (Table 2). In total, there are 143,972 R*XX*L and 6,443 KEN peptides in *S. cerevisiae*, *C. elegans*, *D. melanogaster*, *M. musculus* and *H. sapiens*, while GPS-ARM with the default thresholds predicted 11,417 (∼8%) and 3,932 (∼61%) positive hits for D-boxes and KEN-boxes, respectively (Table 2). Although Motif-D1, Motif-D2, and Motif-KEN generated fewer hits, it can be expected that a large proportion of the real boxes are missed due to their low sensitivity. Also, although Motif-D3 can generate more predicted hits (>3-fold) against GPS-ARM, the results will contain too many false positive hits due to its low specificity (Table 2). Taken together, the prediction performance of GPS-ARM 1.0 is much better than that of the simple short motifs.

**Table 2:** The predicted D-boxes and KEN-boxes in five eukaryotic organisms.

Method		*S. cerevisiae*	*C. elegans*	*D. melanogaster*	*M. musculus*	*H. sapiens*	Total
**RXXL**	Box^a^	12,814	7,555	9,520	51,065	63,018	143,972
	Pro.^b^	4,723	2,519	2,569	13,503	16,477	39,791
**Motif-D1**	Box	118	51	39	216	247	671
	Pro.	117	51	39	213	244	664
**Motif-D2**	Box	817	415	445	1,746	2,123	5,546
	Pro.	732	374	395	1,581	1,916	4,998
**Motif-D3**	Box	3,154	1,849	2,442	12,478	15,138	35,061
	Pro.	2,207	1,192	1,411	7,523	8,963	21,296
**GPS 2.2**	Box	1,104	635	815	4,022	4,841	11,417
	Pro.	958	517	638	3,221	3,832	9,166
**KEN**	Box	1,045	362	387	1,966	2,683	6,443
	Pro.	891	331	340	1,668	2,206	5,436
**Motif-KEN**	Box	143	42	35	159	222	601
	Pro.	138	42	34	157	220	591
**GPS 2.2**	Box	641	227	241	1,191	1,632	3,932
	Pro.	571	216	217	1,052	1,403	3,459

a Box, the number of the predicted boxes;

b Pro., the number of the predicted D-box or KEN-box proteins.

### Functional abundance and diversity of the D-box and KEN-box proteins

It is generally believed that APC/C-mediated degradation plays a predominant role in the cell cycle, especially mitosis [3]–[7], [9]. However, this long-standing view has been challenged by recent discoveries that APC/C is involved in other biological processes [10]–[15]. With GPS-ARM, we predicted thousands of potential D-boxes and KEN-boxes in eukaryotes (Table 2). Although a substantial proportion of the results might not be *bona fide* hits, they still afford a great opportunity to systematically evaluate the functional abundance and distribution of D-box and KEN-box proteins, from the point of view that such prediction results will advance the effort to determine the real box proteins.

From *H. sapiens*, we predicted a total of 4,841 D-boxes in 3,832 proteins and 1,632 KEN-boxes in 1,403 proteins (Table 2). With a hypergeometric distribution [32], we statistically analyzed the enriched biological processes, molecular functions and cellular components with GO annotations for the predicted D-box (Table S2) and KEN-box (Table S3) proteins. Interestingly, for the D-box proteins, the top five most over-represented biological processes are regulation of small GTPase mediated signal transduction (GO:0051056), protein phosphorylation (GO:0006468), regulation of Rho protein signal transduction (GO:0035023), microtubule-based movement (GO:0007018) and axon guidance (GO:0007411) (Table 3). These results suggest that D-box proteins are preferentially implicated in Rho GTPase regulation and axon growth, which is consistent with recently reported experimental observations [10]–[12]. Furthermore, we observed that the GO terms of cell adhesion (GO:0007155) and regulation of glucose transport (GO:0010827) are significantly present (Table 3), and these analyses are also supported by recent studies [13]–[15]. In contrast with the D-box, the functions of the KEN-box proteins are more closely related to the cell cycle and mitosis, although the GO terms for cell adhesion and axon guidance are also enriched (Table 3). Excluding proteins in the training data set did not influence the significance of statistical results for D-boxes (Table S4) and KEN-boxes (Table S5).

**Table 3:** Statistical analysis of the functional abundance and diversity of the D-box and the KEN-box proteins in *H. sapiens*.

Description of GO term	D- or KEN-box		Proteome		E-ratio^c^	*p*-value
	Num.^a^	Per.^b^	Num.	Per.		
***The top 15 most enriched biological processes in D-box proteins***						
Regulation of small GTPase mediated signal transduction (GO:0051056)	70	2.02%	169	0.92%	2.18	1.19E-11
Protein phosphorylation (GO:0006468)	116	3.34%	371	2.03%	1.65	7.27E-09
Regulation of Rho protein signal transduction (GO:0035023)	35	1.01%	72	0.39%	2.56	1.20E-08
Microtubule-based movement (GO:0007018)	39	1.12%	93	0.51%	2.21	2.74E-07
Axon guidance (GO:0007411)	93	2.68%	303	1.66%	1.62	5.54E-07
Intracellular signal transduction (GO:0035556)	82	2.36%	269	1.47%	1.61	3.40E-06
G2/M transition of mitotic cell cycle (GO:0000086)	41	1.18%	110	0.60%	1.96	5.28E-06
Mitotic cell cycle (GO:0000278)	90	2.59%	306	1.67%	1.55	5.95E-06
Intracellular protein kinase cascade (GO:0007243)	35	1.01%	89	0.49%	2.07	6.38E-06
Cell adhesion (GO:0007155)	141	4.06%	546	2.99%	1.36	4.11E-05
Positive regulation of Rho GTPase activity (GO:0032321)	13	0.37%	23	0.13%	2.98	6.84E-05
Peptidyl-serine phosphorylation (GO:0018105)	20	0.58%	45	0.25%	2.34	8.14E-05
Mitotic metaphase/anaphase transition (GO:0007091)	9	0.26%	13	0.07%	3.65	1.08E-04
Nerve growth factor receptor signaling pathway (GO:0048011)	63	1.82%	215	1.18%	1.54	1.56E-04
Regulation of glucose transport (GO:0010827)	15	0.43%	31	0.17%	2.55	1.98E-04
***The top 15 most enriched biological processes in KEN-box proteins***						
Cell cycle (GO:0007049)	71	5.56%	416	2.28%	2.44	1.64E-12
Cell division (GO:0051301)	50	3.92%	267	1.46%	2.68	1.20E-10
Mitotic cell cycle (GO:0000278)	54	4.23%	306	1.67%	2.53	2.21E-10
M phase of mitotic cell cycle (GO:0000087)	26	2.04%	93	0.51%	4.00	5.69E-10
Microtubule-based movement (GO:0007018)	24	1.88%	93	0.51%	3.70	1.48E-08
Flavonoid metabolic process (GO:0009812)	8	0.63%	11	0.06%	10.41	7.55E-08
Mitosis (GO:0007067)	33	2.59%	181	0.99%	2.61	3.22E-07
Golgi to plasma membrane protein transport (GO:0043001)	6	0.47%	7	0.04%	12.27	7.55E-07
Flavone metabolic process (GO:0051552)	5	0.39%	5	0.03%	14.32	1.65E-06
Sulfation (GO:0051923)	6	0.47%	8	0.04%	10.74	2.84E-06
Mitotic prometaphase (GO:0000236)	19	1.49%	85	0.47%	3.20	4.81E-06
Cell adhesion (GO:0007155)	64	5.02%	546	2.99%	1.68	3.08E-05
G2/M transition of mitotic cell cycle (GO:0000086)	20	1.57%	110	0.60%	2.60	6.78E-05
Axon guidance (GO:0007411)	40	3.13%	303	1.66%	1.89	7.58E-05
Protein targeting to lysosome (GO:0006622)	5	0.39%	8	0.04%	8.95	7.72E-05

The top15 most over-represented biological processes are shown.

a the number of proteins annotated;

b the proportion of proteins annotated;

c E-ratio, enrichment ratio, the D-box or KEN-box proportion divided by the human proteome proportion.

To confirm this analysis, we compared the functional diversity of the D-box and KEN-box proteins using the Yates' Chi-square (χ^2^) test [32] (Table S6). Indeed, KEN-box proteins were found to be preferentially involved in mitosis-related processes (Table S6). Taken together, although most of the experimental efforts to date have been performed in an effort to elucidate the regulatory roles of APC/C substrates in the cell cycle and mitosis, our results suggest that the D-box and KEN-box proteins in fact modulate a broad spectrum of biological processes. Again, excluding proteins in the training data set did not influence the significance of final results (Table S7). In this regard, the functional abundance and diversity of newly predicted D-box and KEN-box proteins is similar with total predictions.

### Systematic prediction of mitosis-specific APC/C substrates

The *ab initio* prediction of D-boxes and KEN-boxes inevitably generates a substantial number of false positive hits, because most of these potential boxes may only bind to or be recognized by APC/C *in vitro* and not *in vivo*. It is believed that Cdh1, Cdc20 and APC/C have to co-localize and “kiss” their substrates for interaction to take place in a cell. In this regard, the accurate prediction of *in vivo* APC/C substrates is still a great challenge.

During mitosis, the accumulated evidence suggests that Cdh1, Cdc20 and the core subunits of APC/C complex (e.g., Apc10) localize in various distinct regions, such as the midbody, centrosome, and kinetochore [33]–[36]. Previously, we reported the MiCroKit 3.0 database that contains proteins that localize in the midbody, centrosome and/or kinetochore (microkit proteins) [36]. All of the microkit proteins were experimentally identified with directly corroborating evidence for subcellular localization under fluorescent microscopy [36]. Given the functional importance of the midbody, centrosome and kinetochore in mitosis and co-localization, we hypothesized that the D-boxes and KEN-boxes would likely be enriched in the microkit proteins.

Using the MiCroKit database as a reference and the default thresholds, we predicted a total of 608 potential D-boxes (File S1) and 298 KEN-boxes (File S2) in 421 and 234 proteins, respectively (Table 4). With the hypergeometric test, the statistical results clearly indicated that the D-box and KEN-box proteins are significantly over-represented in the microkit proteins (p≪0.01) (Table 4). In this regard, it is proposed that the midbody, centrosome and kinetochore are potential hotspots of APC/C substrates. The detailed prediction results can also be downloaded at: http://arm.biocuckoo.org/faq.php.

**Table 4:** Statistical results of the potential D-box and KEN-box substrates predicted from the microkit proteins.

Organism	MiCroKit proteins	D-box		E-ratio^c^	*p*-value	KEN-box		E-ratio	*p*-value
		Pro.^a^	Box^b^			Pro.	Box		
***S. cerevisiae***	266	74	93	1.92	5.93E-09	58	70	2.53	1.31E-11
***C. elegans***	95	30	37	2.04	5.24E-05	21	23	3.41	3.55E-07
***D. melanogaster***	112	50	80	2.19	3.19E-09	30	35	3.86	2.67E-11
***M. musculus***	132	52	83	2.00	1.22E-07	24	32	2.83	3.17E-06
***H. sapiens***	677	215	315	1.68	3.25E-16	101	138	2.15	1.51E-13
**Total**	1,282	421	608	1.78	5.50E-36	234	298	2.62	1.18E-42

a Pro., the number of predicted D-box or KEN-box proteins;

b Box, the number of predicted D-boxes or KEN-boxes;

c E-ratio, enrichment ratio, the MiCroKit D-box or KEN-box proportion in comparison with the proteomic proportion.

## Discussion

As one of the most complicated cascades in eukaryotes, the cell cycle is precisely orchestrated by protein biosynthesis, phosphorylation and ubiquitin-dependent degradation in both a temporal and spatial manner [1]–[8]. Identification of APC/C-mediated degradation substrates is crucial for clearly elucidating the molecular mechanisms of the cell cycle. Previous studies suggested that the two APC/C co-activators Cdh1 and Cdc20 are responsible for the recognition of specific targets [16]–[18], [21], [22]. However, a recent analysis using single-particle electron microscopy and NMR spectroscopy reported that a core APC/C subunit of Apc10 also contributes to substrate recognition as a co-receptor of Cdh1 [20]. The efficient identification of APC/C is largely dependent on the discovery of specific boxes or motifs in its substrates. Although a variety of non-consensus motifs have been identified [23]–[25], the D-box and the KEN-box are the two major APC/C recognition motifs [3]–[6], [26]. However, either a too relaxed [5], [17], [18], [21] or too stringent a set of [3]–[5], [16], [22] simple motifs are only of limited use because of their weak predictive power (Table 1).

Previously, the GPS algorithm we developed was mainly used for the prediction of post-translational modification sites in proteins [29]. For the proper usage of the GPS algorithm, the prerequisites are that both the positions of the potentially modified residues and the motif length should be determined and fixed. For example, the lysine residues were regarded as potential pupylation sites in GPS-PUP, while the *pupylation site peptide* was determined as PSP(8, 18) [29]. In this work, we used the core motifs of R*XX*L and KEN in a different manner to determine the APC/C recognition motifs for the D-boxes and KEN-boxes, respectively. The default thresholds stand for the highest *MCC* values of the LOO validations, with an *Ac* of 87.29%, a *Pr* of 82.46%, a *Sn* of 63.51%, a *Sp* of 95.39% and an *MCC* of 0.6463 under the high threshold condition for the D-box, and an *Ac* of 95.00%, a *Pr* of 100.00%, a *Sn* of 93.18%, a *Sp* of 100% and an *MCC* of 0.8858 under the low threshold condition for the KEN-box (Table 1). Since the false positive rates (Type I error in statistics, equal to 1-*Sp*) are quite low due to high *Sp* values, the prediction performance of GPS-ARM is satisfactory. In addition, we collected nine D-boxes and KEN-boxes from recently published articles, while GPS-ARM can predict six of them as positive hits (Table S8).

With the GPS-ARM version 1.0, we directly predicted 11,417 potential D-boxes in 9,166 proteins and 3,932 potential KEN-boxes in 3,459 proteins from five eukaryotic organisms (Table 2). It is proposed that a considerable proportion of the R*XX*L (∼8%) and KEN (∼61%) motifs are real and functional boxes. Since a single predicted protein only contains 1.25 D-box and 1.14 KEN-box, it is concluded that one or two boxes per protein are sufficient for recognition and degradation by APC/C. Beyond the functions of the cell cycle and mitosis, our statistical results indicated that the D-box and KEN-box are involved in additional biological processes (Table 3), and these results are consistent with recently reported experimental observations [10]–[15]. Furthermore, we systematically predicted mitosis-specific APC/C substrates with the localization information from the MiCroKit 3.0 database [36]. Statistical analysis suggested that the D-box and KEN-box proteins are significantly enriched in the midbody, centrosome and kinetochore (Table 4). Taken together, although further improvement should be carried out as new experimental data are available, the GPS-ARM and subsequent analyses provide useful information for further experimental manipulation.

## Supporting Information

File S1 Prediction results of D-boxes in proteins which were localized at MiCroKit (Centrosome, Midbody, and Kinetochore).
(TXT)

File S2 Prediction results of KEN-boxes in proteins which were localized at MiCroKit (Centrosome, Midbody, and Kinetochore).
(TXT)

Table S1 We manually collected 74 experimentally identified D-boxes in 68 unique proteins and 44 experimentally identified KEN-boxes in 42 unique proteins from the scientific literature (PubMed). *a*. UniProt, the UniProt accession numbers of the D-box and KEN-box proteins; *b*. Position, the position of the D-box or KEN-box; *c*. Motif type, the type of known motif that the box follows in. *d*. PMID, the primary references for the known D-boxes or KEN-boxes.
(XLS)

Table S2 The top 15 most enriched biological processes, molecular functions and cellular components of the D-box proteins in *H. sapiens*.
(XLS)

Table S3 The top 15 most enriched biological processes, molecular functions and cellular components of the KEN-box proteins in *H. sapiens*.
(XLS)

Table S4 The top 15 most enriched biological processes, molecular functions and cellular components of newly predicted D-box proteins in *H. sapiens*. The proteins in the training data set were excluded.
(XLS)

Table S5 The top 15 most enriched biological processes, molecular functions and cellular components of newly predicted KEN-box proteins in *H. sapiens*. The proteins in the training data set were excluded.
(XLS)

Table S6 Statistical comparison of the GO terms for the substrates between the predicted KEN-box and D-box proteins in the human proteome. Yates' Chi-square (χ^2^) test was performed (*p*-value<0.05) [32]. The entries with the grey background indicate the Enrichment_ratio ≤1.
(XLS)

Table S7 Statistical comparison of the GO terms for newly predicted KEN-box and D-box proteins in the human proteome. The proteins in the training data set were excluded.
(XLS)

Table S8 From recently published papers, we collected nine D-boxes and KEN-boxes in six proteins. The data set was not used for training. The default parameters were used for the GPS-ARM.
(XLS)
